# Association Between Dysfunctional Parenting Practices and Suspected Gaming Disorder Among Japanese Male Junior High School Students: A Cross-Sectional Study of Parental Assessment

**DOI:** 10.3390/ijerph23060818

**Published:** 2026-06-19

**Authors:** Daisuke Takahara, Misuzu Takahara, Noudéhouénou Credo Adelphe Ahissou, Daisuke Nonaka

**Affiliations:** Graduate School of Health Sciences, University of the Ryukyus, 1076, Ginowan 901-2720, Okinawa, Japan; k228873@cs.u-ryukyu.ac.jp (D.T.); takahara@cs.u-ryukyu.ac.jp (M.T.); adelphahiss@gmail.com (N.C.A.A.)

**Keywords:** gaming disorder, parenting scale, dysfunctional parenting practices, adolescents, parents

## Abstract

**Highlights:**

**Public health relevance—How does this work relate to a public health issue?**

- Adolescent gaming disorder is an escalating global public health concern, highlighting the urgent need to identify modifiable associated factors.

**Public health significance—Why is this work of significance to public health?**

- This study demonstrated that overreactive parenting is independently associated with suspected gaming disorder among Japanese male adolescents.

**Public health implications—What are the key implications or messages for practitioners, policy makers and/or researchers in public health?**

- Parental emotional regulation training may warrant consideration as a component of family-based programs addressing adolescent gaming disorder.

**Abstract:**

The growing prevalence of gaming disorder (GD) in adolescents is a global concern. Despite parents’ critical role in addressing GD, how dysfunctional parenting practices are associated with adolescent GD remains understudied. This study assessed the association between dysfunctional parenting practices and adolescent GD among Japanese male junior high school students. Data were collected in 2024 via web-based, self-administered questionnaires from 300 parents (183 fathers and 117 mothers), each reporting on one male junior high school student. Suspected GD was assessed using a validated parent report measure (i.e., the Gaming Disorder Scale for Parents). Dysfunctional parenting practices were measured using the Parenting Scale, comprising two dimensions: Overreactivity and Laxness. Mean factor scores of Overreactivity and Laxness were compared between the suspected and non-suspected GD groups using a *t*-test. Logistic regression models assessed the association of Overreactivity and Laxness with suspected GD, controlling for covariates. The mean score of Overreactivity was significantly higher in the suspected GD group than in the non-suspected group, whereas that of Laxness was not. After adjustment, overreactive parenting was significantly associated with suspected GD (adjusted odds ratio: 1.89, 95% CI [1.31, 2.74]). This study showed that overreactive parenting was independently and significantly associated with increased odds of suspected GD.

## 1. Introduction

The World Health Organization formally incorporated gaming disorder (GD) into the ICD-11 in 2019 [1]. Gaming disorder, or GD, is characterized by three core elements: impaired control over gaming, increasing priority given to gaming over other activities, and continuation or escalation of gaming despite the occurrence of negative consequences—each of which, in combination, results in serious functional impairment [2]. Prior to ICD-11, the DSM-5 proposed “Internet Gaming Disorder (IGD)” as a condition warranting further study, operationalized through nine criteria [3]. The most fundamental difference between IGD and GD lies in the stringency of diagnostic criteria. To guard against overdiagnosis of ordinary play as pathology, the ICD-11 adopted a higher threshold that places particular weight on significant impairment in daily functioning [4,5]. The prevalence of GD is particularly high in East Asia compared with other regions, suggesting that cultural factors may shape its distribution [6].

The prevalence of GD has increased globally. Although the prevalence of GD varies depending on diagnostic criteria such as ICD-11 and DSM-5 and assessment tools, a meta-analysis reported the pooled prevalence of Internet GD among adolescents to be 7.1% (95% CI [5.6%, 8.5%]) [7]. Among Japanese teenagers and young adults, the prevalence of GD was estimated at 5.1% (95% CI [4.5%, 5.8%]), with substantially higher prevalence in males (7.6%) than females (2.5%) [8]. Adolescents are considered particularly susceptible to problematic gaming behavior, given that the cognitive ability to control behavior and regulate impulses remains underdeveloped [9,10].

Reported risk factors for GD at the individual level include adolescent age, male sex, ADHD, depression, and low self-esteem [11,12]. Regarding gender differences specifically, prior research has pointed to marked disparities—partly attributable to differences in gaming motives, including their function as coping mechanisms for distress [13,14]. While individual characteristics and gender differences interact in complex ways, low family functioning stands out among environmental factors as being strongly associated with GD [15,16]. The family environment is particularly consequential for adolescent development, making family functioning one of the most important risk domains [17]. Indeed, within the range of environmental influences, the family environment has been identified as exerting an especially strong influence on adolescents’ gaming behavior [18,19].

The relationship between family environment and adolescent GD can be understood through three theoretical frameworks. First, self-determination theory holds that psychological well-being depends on the satisfaction of three basic psychological needs: autonomy, competence, and relatedness [20]. When dysfunctional parenting practices chronically frustrate these needs at home, adolescents may turn to gaming as an alternative arena for need satisfaction. Empirical evidence supports this mechanism, showing that adolescents with unmet psychological needs are more prone to problematic gaming behavior [21]. Second, the emotion regulation model complements self-determination theory by suggesting that adolescents who lack effective coping strategies for managing negative affect may resort to gaming as a maladaptive regulatory mechanism [22,23]. This parallels the self-medication hypothesis, originally formulated to explain substance dependence [24]. Applied to behavioral addictions such as GD, the hypothesis suggests that excessive engagement with gaming may function as a form of self-treatment for psychological distress arising from adverse home environments.

Third, from a family systems theory perspective, dysfunctional parenting disrupts family homeostasis, making it more difficult for adolescents to develop adequate self-regulation skills and rendering them vulnerable to the reward mechanisms inherent in gaming [18,25]. Taken together, these three frameworks converge on the quality of parent–child interactions as a key factor associated with adolescents’ risk for GD.

Recent systematic reviews with meta-analyses have documented the substantial influence of parenting-related factors and the family environment on adolescent GD across a wide range of studies [18,26]. Among the specific parenting behaviors examined, parental monitoring [27], parental bonding [28], and parental engagement, including rule setting, have received notable attention [13]. This body of research has considerably broadened our understanding of the family context underlying GD. However, because these studies relied on a broad classification of conceptualized parenting styles, they offer limited guidance on which specific parental behaviors to target for change. Developing effective programs to address GD therefore requires a shift in focus from broad parenting typologies to the concrete behaviors parents actually exhibit in day-to-day discipline situations, that is, parenting practices [29]. Inappropriate and unproductive responses to children’s misbehavior, in particular, have been defined as dysfunctional parenting practices and shown to exert direct adverse effects on children’s mental health [30].

The Parenting Scale (PS) has been widely used to measure such dysfunctional parenting practices in discipline situations [30,31,32]. Although the PS was originally developed to measure the parenting practices of parents of young children [30], subsequent research has confirmed its applicability to a broader demographic, including parents of adolescents [33]. The majority of studies that assessed the factor structure of the PS have shown that the PS is a two-factor model, comprising Overreactivity and Laxness [34,35]. Overreactivity is defined as inappropriate responses to children’s misbehavior characterized by displays of anger, meanness, and irritability [30]—reflecting, in essence, a breakdown in parental emotional self-regulation [36]. Laxness, by contrast, is associated with permissive and inconsistent parenting, referring to attitudes where parents give in to their children, allow rules to go unenforced, or provide positive consequences for misbehavior [30,35]. Empirical studies examining how these two specific dimensions of parenting practices are associated with suspected GD in adolescents remain limited.

The objective of the present study was to assess the association between dysfunctional parenting practices, measured by the PS (i.e., Overreactivity and Laxness), and suspected GD of Japanese male junior high school students. Suspected adolescent GD was measured using a parent-rated screening scale [37,38], which enables evaluation of gaming behavior from the parental perspective. Drawing on the theoretical frameworks outlined above, we formulated two directional hypotheses. First, higher parental Overreactivity would be positively associated with suspected GD, given that overreactive parenting is likely to frustrate children’s basic psychological needs, induce distress, and may be associated with escapist engagement in gaming as a form of compensatory behavior. Second, higher parental Laxness would also be positively associated with suspected GD, because inconsistent discipline may hinder the development of the self-regulation skills needed for adolescents to control their gaming. The findings of the present study may help to design a prevention program for young students with GD.

## 2. Materials and Methods

### 2.1. Study Participants

This cross-sectional study was conducted with 300 parents (183 fathers and 117 mothers, each reporting on one distinct male junior high school student) who live in an urban area of Tokyo. The inclusion criteria for parents were as follows: (1) guardianship of a male junior high school child, (2) living in an urban area of Tokyo, and (3) registration with an Internet research company (MyVoice Communications, Inc.). The exclusion criteria were (1) parents not living together with the schoolchild and (2) parents whose schoolchild does not play games. The present study focused exclusively on male junior high school students for two reasons. First, male adolescents are known to have a substantially higher prevalence of GD than female adolescents [8]. Second, within a constrained sample size, focusing on a homogeneous, high-risk population of active male gamers was deemed methodologically efficient for detecting meaningful associations while controlling for sex-based confounding. By excluding non-gaming children, this study intentionally focused on a high-risk population of active gamers. Furthermore, parents were surveyed rather than adolescents directly because data collection among adolescents in Japan presents considerable practical and ethical challenges, including the need for school cooperation consent.

Although the present study did not collect the age information of the schoolchildren, almost all junior high school students are considered to be aged between 12 and 15 (i.e., first grade: 12–13; second grade: 13–14; and third grade: 14–15), as repeating a year is very rare in Japanese junior high schools. The sample size of 300 was determined a priori to ensure robust validation of the PS. Following the established rule of thumb for factor analysis, which recommends a minimum participant-to-item ratio of 10:1 [39,40], a sample of 300 was deemed optimal for the 30-item PS.

### 2.2. Data Collection

Data were collected between 15 and 20 February 2024. The Internet research company sent an invitation email that included the URL of an online questionnaire to 5009 company registrants (selected from an active pool of 10,049 monitors prescreened for parental age [30–79 years], residence in Tokyo, and having a junior high school child) who potentially met the inclusion criteria. To encourage honest self-identification, the survey was administered in complete anonymity. The research company employs quality-control measures to ensure that all registrants are verified individuals. Among them, 1067 accessed the URL, and 1040 responded to screening questions (i.e., consent to participate, residence in Tokyo, co-living family structure, and having a male junior high school student who actively plays internet games) to verify the inclusion and exclusion criteria. As a result, 330 met the criteria and completed the questionnaire. Finally, 30 respondents whose responses were considered invalid were excluded: those who selected the same response option for all questions and those who completed the survey in an implausible time frame (i.e., less than approximately 200 s or more than approximately 90 min). On participation, respondents were given a reward incentive of up to 55 points worth 55 Japanese yen (approximately 0.4 US dollars at the time of the survey).

### 2.3. Variables and Measurements

The primary outcome of the present study was suspected adolescent GD measured by the Gaming Disorder Scale for Parents (GADIS-P). The GADIS-P was selected because it is currently the only parent-reported instrument strictly developed based on the ICD-11 diagnostic criteria for GD [37]. The linguistic validity of a Japanese version has been confirmed [38]. The GADIS-P comprises nine symptom items rated on a five-point Likert scale ranging from “strongly disagree” to “strongly agree”, and in the Japanese version, one timing item is rated by five ordered response options: “not at all”, “only on a single day”, “several days to one month”, “one to several months”, and “almost daily”. A score of 0 is given to “strongly disagree” and “not at all”, whereas a score of 4 is given to “strongly agree” and “almost daily.” The GADIS-P is a two-factor model: the first factor is “cognitive behavioral symptoms”, measured by four symptom items, and the second factor is “negative consequences”, measured by five symptom items. A child is considered suspected of GD when the following conditions are met: (1) the score of the first factor is 10 or above, (2) the score of the second factor is 6 or above, and (3) the score of the one timing item is 2 or higher.

Specifically, the optimal cut-offs for the two symptom factors were empirically derived using receiver operating characteristic (ROC) curve analyses, maximizing Youden’s index against classifications based on an established GD screening measure in the original validation study [37]. These thresholds align with the cut-offs used in the adolescent self-report version (GADIS-A), supporting the comparability of assessments across informants [37]. Furthermore, the timing item evaluates the ICD-11 duration criterion (i.e., symptoms persisting for at least 12 months). During the linguistic validation of the Japanese version, the original four-point scale for the timing item was refined into a five-point scale to reduce linguistic ambiguity regarding symptom duration, and a score of 2 (“several days to one month”) or higher was established to correspond to the original clinical threshold [38]. Although the GADIS-P was developed based on the ICD-11 diagnostic criteria and demonstrates excellent discriminatory power and criterion validity [37], parental assessments may not fully align with clinical diagnoses. Therefore, the term “suspected GD” is used deliberately throughout this manuscript to reflect the screening, rather than diagnostic, nature of the instrument.

The main exposure variable was the dysfunctional parenting practices measured by the PS. The PS was selected because it specifically operationalizes the theoretical constructs of Overreactivity and Laxness, which are theoretically linked to adolescent GD through emotional regulation and systemic family frameworks. A Japanese version of the PS, which underwent rigorous cross-cultural adaptation and linguistic validation, was developed and validated for the use of parents of young children [41].

The covariate variables were adolescent behavioral and emotional difficulties measured by the Strengths and Difficulties Questionnaire (SDQ) [42,43,44]; parents’ psychological distress measured by the Kessler Psychological Distress Scale (K10) as an indicator of parental mental health [45,46]; socio-economic and demographic characteristics of parents (i.e., gender, age, working style, educational background, and annual household income as indicators of socioeconomic status and number of children living together as an indicator of family structure); and characteristics of the adolescents and their gaming (i.e., school grade, age when they started gaming, excessive in-game spending, family rules about gaming, parental controls by monitoring function, and number of friends).

### 2.4. Validation of the Parenting Scale

The psychometric properties of the Japanese version of the PS were examined in the present sample through a series of validation analyses. First, the factorial structure of the PS was evaluated using both confirmatory factor analysis (CFA) and exploratory factor analysis (EFA) to verify and refine the two-factor model comprising Overreactivity and Laxness. Second, discriminant validity between the two PS factors was examined. Third, evidence of convergent validity was assessed by examining the associations between PS factor scores and two external criterion variables using Spearman’s rank correlation: (1) the total difficulties score of the SDQ, based on the rationale that parents’ responses to children’s misbehavior are likely influenced by the child’s behavioral and emotional difficulties; and (2) the K10 score, based on the rationale that parental mental health status similarly influences parenting behavior. Positive correlations between PS factor scores and both criterion variables were hypothesized.

### 2.5. Statistical Analysis

Prior to conducting inferential analyses, the distributions of continuous variables were assessed for normality using the Kolmogorov–Smirnov test, skewness and kurtosis indices, and visual inspection of histograms. Independent-sample *t*-tests were used to examine bivariate associations between the Parenting Scale factors and the outcome variable. Fisher’s exact test was used to assess associations between the categorical covariate variables and the outcome variable. The Mann–Whitney U test was used to assess associations between the non-normally distributed continuous covariates and the outcome variable. Appropriate effect sizes were calculated: Cohen’s d for parametric continuous variables (Parenting Scale factors), Cramer’s *V* for categorical variables (Fisher’s exact test), and effect size *r* for nonparametric continuous variables (Mann–Whitney U test). The significance level was set at *p* < 0.05 (two-tailed).

Binary logistic regression was conducted to examine two models in a multivariate analysis. Model 1 included the exposure variables along with the covariate variables representing parental characteristics, such as gender, age, working style, educational attainment, household income, number of children living together, and parents’ mental health (K10). In addition to these, Model 2 included covariates representing child characteristics, such as school grade, number of friends, and child difficulties (SDQ), and gaming-related characteristics such as age when they started gaming, excessive in-game spending, and family rules about gaming. Prior to logistic regression analyses, multicollinearity among all independent variables was assessed using variance inflation factors, with values exceeding 10 indicating problematic collinearity. Model fit was evaluated using the Nagelkerke R^2^ and the Hosmer–Lemeshow goodness-of-fit test for both models. Effect sizes for logistic regression are reported as adjusted odds ratios (aORs) with 95% confidence intervals (CIs).

To explore potential developmental moderation effects, a sensitivity analysis was conducted by stratifying based on students’ school grade in Model 2. All analyses were performed using IBM SPSS Statistics version 22, while the forest plot illustrating odds ratios from Model 2 was produced using Stata 17.

### 2.6. Ethical Considerations

Given the anonymous and non-interventional nature of the online survey, the Ethics Committee waived the requirement for formal written informed consent in favor of electronic consent. A detailed explanation of the study’s purpose, data handling, and privacy protections was provided online to all potential participants. Participants confirmed their voluntary participation by checking the designated “consent” box before accessing the questionnaire. This study was approved by the Ethics Committee for Medical and Health Research Involving Human Subjects of the University of the Ryukyus, Japan (permit number: 23-2236-00-00-00, 17 January 2024).

## 3. Results

### 3.1. Psychometric Properties of the Measurements

A CFA confirmed the two-factor structure (9 items: 5 for Overreactivity and 4 for Laxness) of the Japanese version of the Parenting Scale (PS). The goodness-of-fit indices demonstrated an adequate fit to the data: CFI = 0.923, SRMR = 0.077, and RMSEA = 0.085 (90% CI [0.064, 0.106]) [47]. Internal consistency was adequate for Overreactivity (Cronbach’s α = 0.80) and Laxness (Cronbach’s α = 0.72). Discriminant validity was supported by the lack of a significant correlation between the two factors (*ρ* = 0.02, *p* = 0.757). Convergent validity was established through significant positive correlations between the PS factors and criterion variables (Overreactivity with SDQ: *ρ* = 0.29 and K10: *ρ* = 0.23; Laxness with SDQ: *ρ* = 0.34 and K10: *ρ* = 0.26; all the *p*-values less than 0.01). The internal consistency of the GADIS-P was strong (Cronbach’s α = 0.92 for the total core symptoms, 0.86 for cognitive-behavioral symptoms, and 0.90 for negative consequences).

### 3.2. Characteristics of Study Participants

The sociodemographic and gaming-related characteristics of the participants are detailed in Table 1. Briefly, the sample was predominantly characterized by highly educated, middle- to high-income parents residing in urban Tokyo. Regarding adolescents, a vast majority had initiated digital gameplay at or before age 12, and approximately two-thirds were subject to specific family rules regarding gaming.

### 3.3. Bivariate Associations

The mean factor score of Overreactivity was significantly higher in the suspected GD group than in the non-suspected GD group (0.56 [*SD* = 0.99] vs. −0.03 [*SD* = 0.97]) (Table 2). In contrast, there was no significant difference in the mean score of Laxness between the two groups (−0.02 [*SD* = 0.99] vs. 0.03 [*SD* = 1.00]). Among the covariate variables, suspected GD was significantly associated with higher scores on the SDQ total difficulties (*p* < 0.001, *r* = 0.26) and parental K10 (*p* = 0.008, *r* = 0.15). Having few or no friends was also significantly associated with suspected GD (*p* = 0.037, Cramer’s *V* = 0.127). Other sociodemographic and gaming characteristics were not significantly associated with the outcome (Table 2).

### 3.4. Multivariate Associations of Suspected Gaming Disorder

Variance inflation factors for all independent variables ranged from 1.06 to 2.31, indicating that multicollinearity was not a concern. Model fit was adequate for models 1 and 2 (Table 3). The bivariate logistic regression analysis showed that suspected GD was significantly associated with higher levels of Overreactivity (cOR = 1.82, 95% CI [1.35, 2.44]). In Model 1, which adjusted for parental characteristics, Overreactivity remained significantly associated with suspected GD (adjusted odds ratio [aOR] = 1.94, 95% CI [1.38, 2.73]). In Model 2, which additionally adjusted for child and gaming characteristics, the multivariate logistic regression analysis showed a similar significant association (aOR = 1.89, 95% CI [1.31, 2.74]). Unlike Overreactivity, logistic regression analyses did not show a significant association between Laxness and suspected GD in any model (aOR = 0.85, 95% CI [0.60, 1.19] in Model 2). The adjusted odds ratios and 95% CIs from Model 2 are shown in the forest plot in Supplementary Figure S1.

### 3.5. Sensitivity Analyses

To explore potential developmental moderation effects, the fully adjusted logistic regression model was stratified by the students’ school grades. Overreactivity was significantly associated with suspected GD among first-year (aOR = 2.52, 95% CI [1.09, 5.79]) and second-year junior high school students (aOR = 2.03, 95% CI [1.12, 3.66]). However, this association was not statistically significant among third-year students (aOR = 1.23, 95% CI [0.58, 2.63]). Laxness was not significantly associated with suspected GD in any grade stratum (first-year: aOR = 0.60, 95% CI [0.29, 1.24]; Table 4).

## 4. Discussion

The present study found that parental Overreactivity was associated with suspected GD among Japanese male junior high school students. This finding is consistent with studies in China, South Korea, Taiwan, India, Germany, France, Spain, Sweden, Turkey, and Australia, all reporting that dysfunctional parenting—including psychological control and overinvolvement—is linked to a greater risk of adolescent GD [26]. From the perspective of self-determination theory [20], parental Overreactivity—marked by emotional outbursts and harsh discipline—may frustrate adolescents’ basic needs for autonomy and relatedness [48] and is associated with serious psychological distress, including anxiety and depression [49,50].

Building on the self-medication hypothesis [24], children who experience such distress at home may turn to excessive gaming as a means of coping with or escaping it, which may in turn raise GD [22,23]. The restriction of the sample to male adolescents is relevant in this context: compared with females, males rely more heavily on escapist gaming as a coping strategy for distress [14], which may partly explain the observed association between parental Overreactivity and suspected GD.

Additionally, child difficulties (higher quartiles of the SDQ total difficulties score) showed the strongest association with suspected GD among all variables in the multivariate models. This indicates that pre-existing psychosocial difficulties in the child are a major correlate of GD and raises the possibility of bidirectional influences within the parent–child relationship. From a family systems perspective [13,18], parenting a child who already exhibits behavioral problems or early gaming-related difficulties may erode parental tolerance and elicit overreactive responses. Nonetheless, the present study’s focus on parental Overreactivity is clinically meaningful. Although a child’s temperament or pre-existing difficulties are hard to modify directly, overreactive parenting—reflecting a transient breakdown in emotional self-regulation during discipline—responds well to targeted intervention [36].

Unlike parental Overreactivity, parental Laxness was not significantly associated with suspected GD. Laxness, as measured by the Parenting Scale, does not reflect deliberate autonomy support but rather a failure to enforce consistent rules and follow through on discipline [30]. The absence of a significant association with Laxness suggests that, for male junior high school students in contemporary Japan, parental emotional reactivity and harsh responses may be a more psychologically salient source of stress—and more strongly associated with escapist gaming—than parental leniency. This pattern is broadly consistent with recent findings from East Asian societies characterized by high academic pressure and conformist norms, such as China and South Korea [51,52,53,54]. Meanwhile, caution is warranted in transferring findings from these settings, given the subtle but meaningful cultural differences in parent–child dynamics. The absence of significant associations between suspected GD and gaming-related covariates (e.g., family rules, parental controls, and early gaming onset) should be interpreted with caution. Because the present sample was deliberately restricted to active adolescent gamers, restricted variance in these gaming-related variables likely attenuated the observed associations.

The sensitivity analyses further suggested that the influence of parenting behavior may vary with the adolescent’s developmental stage. The association between parental Overreactivity and suspected GD was stronger among first-year (aOR = 2.52) and second-year (aOR = 2.03) junior high school students than among third-year students (aOR = 1.23). This pattern may reflect a developmental shift across mid-adolescence. As adolescents grow older and gain greater autonomy outside the home, the direct influence of parental discipline diminishes while peer relationships become increasingly important in shaping behavior [55,56]. In East Asian contexts, academic stress from competitive educational environments may further shape family dynamics and, in older adolescents, may eventually outweigh parental influences in shaping behavior.

The prevalence of suspected GD in the present study (24.0%) was substantially higher than the pooled prevalence reported in a recent meta-analysis (7.1%) [7]. Two main factors may explain this discrepancy. First, the sample was restricted to parents of male junior high school students who actively play video games; male adolescents are a well-established high-risk group for GD [8], and non-gamers were excluded by design, which inevitably elevated the estimated prevalence. Second, GD was assessed exclusively through parental report, and prior research has consistently shown that parents tend to rate their children’s symptoms as more severe than children would report [57,58]. The reliance on a screening measure and the potential for shared method bias, given that all variables were rated by the same informant, may have further inflated the prevalence estimate.

Although the cross-sectional design of the present study does not permit causal inferences, the findings have important public health implications for prevention and intervention. Prior studies have shown that approaches aimed at improving parental emotional self-regulation effectively reduce overreactive discipline [36]. Future longitudinal studies may help determine whether the association between parental Overreactivity and suspected adolescent GD reflects a directional pathway, reverse causation (in which children’s gaming-related difficulties elicit overreactive parenting), or both. In addition, multi-informant designs that incorporate adolescent self-report and clinical interviews would further address the limitations of single-informant screening data [55,57].

The present study has several limitations that should be considered when interpreting the findings. First, the cross-sectional design does not allow the direction of causal relationships between variables to be determined. Although overreactive parenting might have caused suspected GD in children, the reverse is equally plausible: children’s gaming-related symptoms and associated behavioral difficulties may elicit overreactive and inconsistent parenting responses.

Second, all variables in the present study were obtained mainly from parental self-reports. The use of a single informant increases the risk of shared method bias and perceptual bias and may have led to an overestimation of the observed associations. Furthermore, parental psychological distress, as measured by the K10, may have influenced parents’ subjective ratings of both their children’s behavior and their own parenting practices. To partially mitigate this, the K10 score was included as a covariate in all multivariate models. Additionally, the GADIS-P was a validated parent-report instrument with strong criterion performance [37].

Third, the sample was drawn from an online panel of an Internet research company and restricted to parents of male, gaming adolescents residing in urban Tokyo, which may have introduced self-selection and technology bias by overrepresenting digitally engaged families. The restriction was a deliberate methodological choice—to focus on a homogeneous, high-risk group and strengthen internal validity—but it limits generalizability to female adolescents, non-gamers, and rural populations. Although the response incentive was minimal, its potential influence on response quality cannot be entirely ruled out; we therefore applied a pre-specified, multi-criterion data-cleaning procedure (excluding straight-line responses and implausibly short or long completion times) to minimize careless responding. Exact ages of the students were not collected, but school grade served as a reliable proxy because grade repetition is very rare in Japanese junior high schools.

Fourth, suspected GD was assessed using a parental screening measure (GADIS-P) rather than a clinical diagnosis based on a structured interview aligned with ICD-11 diagnostic criteria for GD. Parental ratings do not always align with clinical assessments, and parents tend to overestimate the severity of their children’s symptoms relative to the children’s own self-reports [57,58]. We mitigate this concern by referring to the variable consistently as “suspected” GD and by using an instrument with documented discriminatory performance in its original validation (AUC > 0.92; sensitivities 87.0–92.8%; specificities 84.1–86.1%) [37].

Fifth, Model 2 included a relatively large number of covariates relative to the sample size (*n* = 300 overall; suspected GD group *n* = 72), and the risk of model overfitting and unstable estimates cannot be entirely excluded. With more than 15 covariates included in Model 2, the events-per-variable ratio falls below the conventional threshold of 10 [59], suggesting that some estimates—particularly for less common exposures—may be unstable. Future research should address this limitation by employing larger sample sizes to ensure adequate statistical power and enable robust evaluation of multiple covariates.

## 5. Conclusions

This study showed that parental Overreactivity was independently and significantly associated with increased odds of suspected GD among Japanese male junior high school students, with the association being particularly pronounced among younger adolescents in their first and second years. Longitudinal studies are needed to clarify the directionality of this association, including the possibility of reverse causation, before formulating definitive clinical interventions.

## Figures and Tables

**Table 1:** Characteristics of study participants (*n* = 300).

Characteristics	Categories	*n*	%
Parental characteristics			
Gender	Male	183	61.0
	Female	117	39.0
Age	30s–40s	165	55.0
	50s or older	135	45.0
Working style	Full-time employee	186	62.0
	Part-time/temporary/other	114	38.0
Educational attainment	University graduate or above	188	62.7
	Lower than a university graduate	112	37.3
Annual household income	<4 million Yen	33	11.0
	4–8 million Yen	101	33.7
	>8 million Yen	166	55.3
Number of children living together	1 child	76	25.3
	2 children	163	54.3
	≥3 children	61	20.3
Child/gaming characteristics			
School grade	1st grade	89	29.7
	2nd grade	114	38.0
	3rd grade	97	32.3
Number of friends	Many/some	183	61.0
	Few/none	117	39.0
Age at which the child started gaming	≤12 years old	222	74.0
	13–15 years old	78	26.0
Excessive in-game spending	Experienced	28	9.3
	Not experienced/unknown	272	90.7
Family rules about gaming	Existing	202	67.3
	Not existing/unknown	98	32.7
Parental controls (monitoring function)	Utilized	113	37.7
	Not utilized/unknown	187	62.3

**Table 2:** Bivariate associations of suspected gaming disorder with exposure and covariate variables (*n* = 300).

Variables	Non-Suspect (*n* = 228)		Suspect (*n* = 72)		*p*	Effect Size
	*n*	%	*n*	%		
The Parenting Scale						
Overreactivity: mean (standard deviation)	−0.03 (0.97)		0.56 (0.99)		<0.001 ^a^	*d* = 0.61 ^d^
Laxness: mean (standard deviation)	0.03 (1.00)		−0.02 (0.99)		0.763 ^a^	*d* = −0.05 ^d^
Gender					0.452 ^b^	*V* = 0.015 ^e^
Male	140	61.4	43	59.7		
Female	88	38.6	29	40.3		
Age					0.893 ^b^	*V* = 0.009 ^e^
30s–40s	126	55.3	39	54.2		
50s or older	102	44.7	33	45.8		
Working style					0.404 ^b^	*V* = 0.054 ^e^
Full-time employee	138	60.5	48	66.7		
Part-time/temporary/other	90	39.5	24	33.3		
Educational attainment					0.781 ^b^	*V* = 0.018 ^e^
University graduate or above	144	63.2	44	61.1		
Lower than a university graduate	84	36.8	28	38.9		
Annual household income					0.152 ^b^	*V* = 0.112 ^e^
<4 million Yen	21	9.2	12	16.7		
4–8 million Yen	81	35.5	20	27.8		
>8 million Yen	126	55.3	40	55.6		
Number of children					0.872 ^b^	*V* = 0.030 ^e^
1	59	25.9	17	23.6		
2	124	54.4	39	54.2		
≥3 children	45	19.7	16	22.2		
School grade					0.619 ^b^	*V* = 0.059 ^e^
1st grade	69	30.3	20	27.8		
2nd grade	83	36.4	31	43.1		
3rd grade	76	33.3	21	29.2		
Number of friends					0.037 ^b^	*V* = 0.127 ^e^
Many/some	147	64.5	36	50.0		
Few/None	81	35.5	36	50.0		
Age at which children started gaming					0.283 ^b^	*V* = 0.066 ^e^
≤12 years old	165	72.4	57	79.2		
13–15 years old	63	27.6	15	20.8		
Excessive in-game spending					0.351 ^b^	*V* = 0.061 ^e^
Experienced	19	8.3	9	12.5		
Not experienced/unknown	209	91.7	63	87.5		
Family rules about gaming					0.198 ^b^	*V* = 0.075 ^e^
Existing	158	69.3	44	61.1		
Not existing/unknown	70	30.7	28	38.9		
Parental controls (monitoring function)					1.000 ^b^	*V* = 0.002 ^e^
Utilized	86	37.7	27	37.5		
Not utilized/unknown	142	62.3	45	62.5		
Parental mental health (K10 score)						
Median (interquartile range)	15 (12)		19 (17)		0.008 ^c^	*r* = 0.15 ^f^
Child difficulties (total difficulties score of SDQ)						
Median (interquartile range)	9 (8)		13 (8)		<0.001 ^c^	*r* = 0.26 ^f^

^a^ Independent-sample *t*-test. ^b^ Fisher’s exact test. ^c^ Mann–Whitney U test. Effect sizes: ^d^ Cohen’s d. ^e^ Cramer’s V. ^f^ Effect size r. SDQ = Strengths and Difficulties Questionnaire; K10 = Kessler Psychological Distress Scale.

**Table 3:** Multivariable logistic regression of association with suspected GD (*n* = 300).

Variable	Category	Model 1		Model 2	
		aOR ^a^	95% CI ^b^	aOR ^a^	95% CI ^b^
The Parenting Scale (factor scores)					
Overreactivity	—	1.94	[1.38, 2.73]	1.89	[1.31, 2.74]
Laxness	—	1.00	[0.74, 1.36]	0.85	[0.60, 1.19]
Parental characteristics					
Gender	Male (ref)	1	—	1	—
	Female	1.08	[0.46, 2.54]	1.14	[0.45, 2.86]
Age	30s–40s (ref)	1	—	1	—
	50s or older	1.26	[0.70, 2.29]	1.34	[0.70, 2.57]
Working style	Full-time employee (ref)	1	—	1	—
	Part-time/temporary/other	0.57	[0.24, 1.32]	0.68	[0.28, 1.67]
Educational attainment	University graduate or higher (ref)	1	—	1	—
	Lower than a university graduate	1.13	[0.59, 2.18]	1.13	[0.56, 2.26]
Annual household income	<4 million Yen (ref)	1	—	1	—
	4–8 million Yen	0.53	[0.21, 1.33]	0.60	[0.22, 1.62]
	>8 million Yen	0.67	[0.27, 1.64]	0.95	[0.36, 2.52]
Number of children	1 child (ref)	1	—	1	—
	2 children	1.10	[0.56, 2.20]	1.27	[0.61, 2.62]
	≥3 children	1.36	[0.59, 3.17]	1.77	[0.72, 4.36]
Parental mental health ^c^	Lowest quartile (ref)	1	—	1	—
	Second quartile	2.28	[0.97, 5.34]	2.45	[0.99, 6.04]
	Third quartile	1.39	[0.60, 3.23]	1.01	[0.41, 2.48]
	Highest quartile	2.07	[0.88, 4.88]	1.52	[0.60, 3.86]
Child/gaming characteristics					
School grade	1st grade (ref)	—	—	1	—
	2nd grade	—	—	1.09	[0.51, 2.35]
	3rd grade	—	—	0.86	[0.37, 1.95]
Number of friends	Many/some (ref)	—	—	1	—
	Few/none	—	—	1.44	[0.75, 2.79]
Age when started gaming	Junior high school (ref)	—	—	1	—
	Elementary school or earlier	—	—	1.39	[0.65, 2.94]
Excessive in-game spending	Not experienced/unknown (ref)	—	—	1	—
	Experienced	—	—	2.04	[0.78, 5.34]
Family rules about gaming	Rules exist (ref)	—	—	1	—
	No rules/unknown	—	—	1.64	[0.85, 3.17]
Parental controls	Utilized (ref)	—	—	1	—
	Not utilized/unknown	—	—	0.97	[0.50, 1.89]
Child difficulties ^d^	Lowest quartile (ref)	—	—	1	—
	Second quartile	—	—	1.86	[0.67, 5.20]
	Third quartile	—	—	5.24	[1.93, 14.24]
	Highest quartile	—	—	4.02	[1.28, 12.55]

Model 1: Nagelkerke R^2^ = 0.135; Hosmer–Lemeshow: χ^2^(8) = 12.936, *p* = 0.114. Model 2: Nagelkerke R^2^ = 0.233; Hosmer–Lemeshow: χ^2^(8) = 12.442, *p* = 0.133. ^a^ Adjusted odds ratio. ^b^ 95% confidence interval. ^c^ K10 score. ^d^ Total difficulties score of SDQ.

**Table 4:** Sensitivity analysis: multivariable logistic regression of suspected gaming disorder stratified by school grade (*n* = 300).

School Grade	*n*	Overreactivity		Laxness	
		aOR ^a^	95% CI ^b^	aOR ^a^	95% CI ^b^
1st grade	89	2.52	[1.09, 5.79]	0.60	[0.29, 1.24]
2nd grade	114	2.03	[1.12, 3.66]	1.45	[0.80, 2.61]
3rd grade	97	1.23	[0.58, 2.63]	0.62	[0.28, 1.35]

^a^ Adjusted odds ratio. ^b^ 95% confidence interval.

## Data Availability

The data presented in this study are available upon request from the corresponding author due to ethical reasons.

## Supplementary Materials

The following supporting information can be downloaded at: https://www.mdpi.com/article/10.3390/ijerph23060818/s1, Figure S1: Forest Plot of Adjusted Odds Ratios (Model 2): Association Between Parenting Practices and Suspected Gaming Disorder (n = 300).
