# Supplementary material for: Association Between Dysfunctional Parenting Practices and Suspected Gaming Disorder Among Japanese Male Junior High School Students: A Cross-Sectional Study of Parental Assessment
Source: Int J Environ Res Public Health. 2026 Jun 19;23(6):818. doi: 10.3390/ijerph23060818 (PMC13299178; doi:10.3390/ijerph23060818)
Supplement: Supplementary file 1 [file ijerph-23-00818-s001.zip › ijerph-4319708-supplementary.pdf]

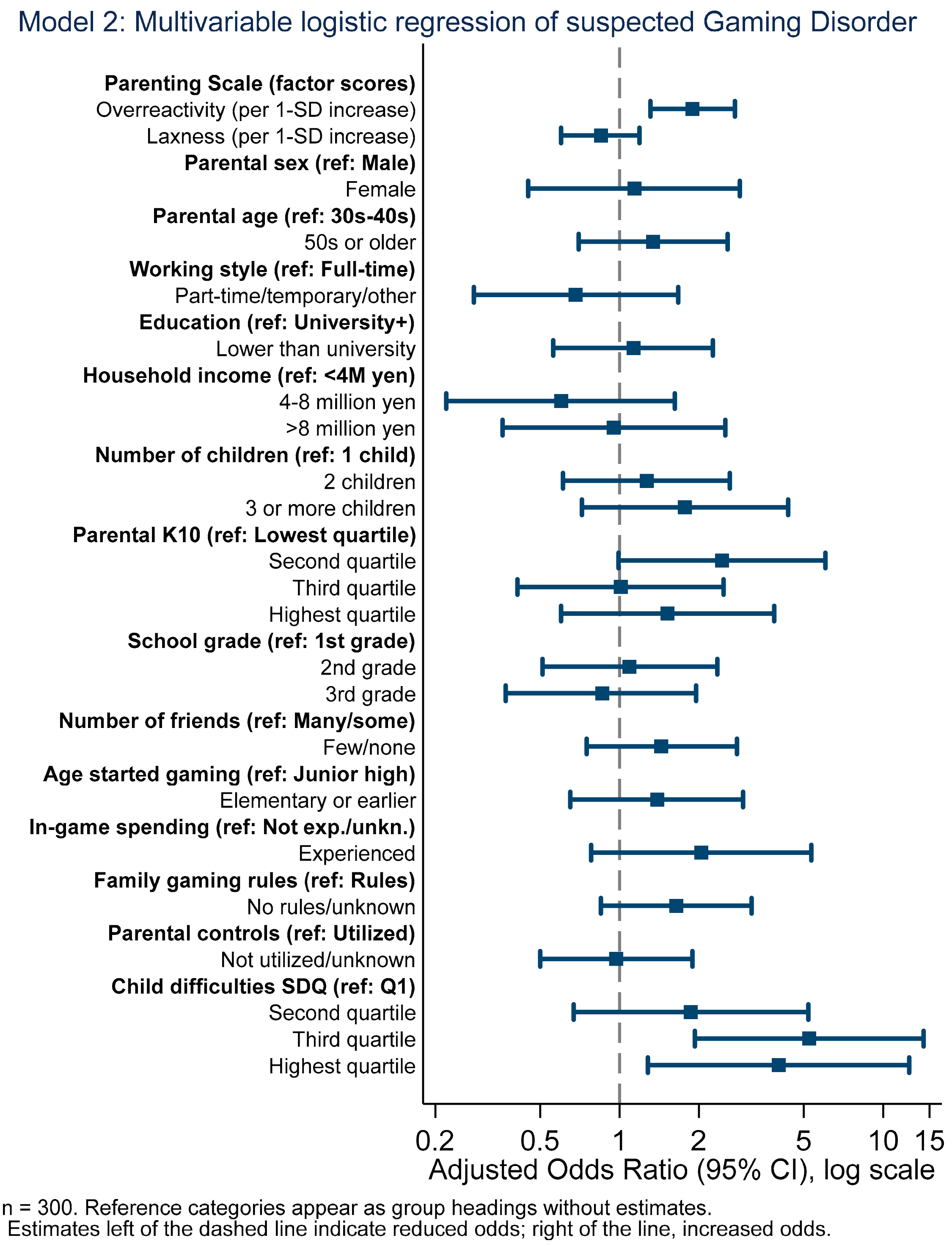


Supplementary Figure S1. Forest Plot of Adjusted Odds Ratios (Model 2): Association Between Parenting Practices and Suspected Gaming Disorder ($n$ = 300).
